# The response of leptin, interleukin-6 and fat oxidation to feeding in weight-losing patients with pancreatic cancer

**DOI:** 10.1038/sj.bjc.6601712

**Published:** 2004-03-02

**Authors:** M D Barber, D C McMillan, A M Wallace, J A Ross, T Preston, K C H Fearon

**Affiliations:** 1 University Department of Surgery, Royal Infirmary, Edinburgh EH3 9YW, UK; 2 University Department of Surgery, Royal Infirmary, Glasgow G31 2ER, UK; 3 Department of Clinical Biochemistry, Royal Infirmary, Glasgow G31 2ER, UK; 4 Isotope Biochemistry Laboratory, Scottish Universities Environmental Research Centre, East Kilbride, Glasgow G75 OQF, UK

**Keywords:** leptin, interleukin-6, insulin, cortisol, fat oxidation, feeding, weight loss, pancreatic cancer

## Abstract

At baseline, weight-losing pancreatic cancer patients (*n*=7) had lower leptin (*P*<0.05) but higher cortisol, interleukin-6, resting energy expenditure and fat oxidation than healthy subjects (*n*=6, *P*<0.05). Over a 4 h feeding period, the areas under the curve for glucose, cortisol and interleukin-6 were greater (*P*<0.05), but less for leptin in the cancer group (*P*<0.05). Therefore, it would appear that low leptin concentrations, increased fat oxidation and insulin resistance are associated with increased concentrations of cortisol and interleukin-6 in weight-losing patients with pancreatic cancer.

The discovery of leptin, an adipocyte-secreted protein that acts on the central nervous system to regulate body weight in animals (Pelleymounter *et al*, 1995), has stimulated interest in the role of leptin in humans both in the obese and cachectic states. Evidence, in animals, suggests that circulating leptin concentrations regulate energy intake centrally (primarily through appetite) and expenditure (primarily through substrate oxidation) by a control feedback loop involving neuropeptide Y (Inui, 1999). Evidence, in humans, indicates that the major role of leptin appears in the adaptation to reduced energy intake and reduced body fat stores (Havel, 2002).

In studies of cachectic cancer patients, the role of leptin appears more complex since the fall in leptin concentrations which accompanies a loss of body fat appears not to be associated with an improvement in energy balance (Simons *et al*, 1997; Wallace *et al*, 1998). Little is known about the regulation of leptin production during feeding and its effect on fat oxidation in humans. However, recent studies have indicated that increased leptin concentrations are associated with increased fat oxidation (Havel, 2002).

It is now recognised that the presence of a systemic inflammatory response is associated with an increase in substrate oxidation and resting energy expenditure in patients with chronic disease states such as cancer (Fearon *et al*, 1999; Kotler, 2000). In particular, the pro-inflammatory cytokine interleukin-6 is likely to be important in this catabolic process since it is known to act on the central nervous system to reduce appetite (Havel, 2002), results in increased substrate oxidation when infused (Stouthard *et al*, 1995) and is recognised to be associated with weight loss in cancer patients (Fearon *et al*, 1991; Scott *et al*, 1996).

It is of interest that interleukin-6 shares structural and some functional similarities to leptin. Both are released in response to injury at a similar time (Wallace *et al*, 2000), and are capable of activating the same hypothalamic receptor (Inui, 1999), and therefore interleukin-6 might mimic the anorexogenic effect of leptin. This concept is consistent with the observations that intracerebroventricular infusion of physiological doses of interleukin-6 in rodents has been shown to result in reduction of fat mass without inducing an acute-phase protein response (Wallenius *et al*, 2002).

It has long been recognised that following an overnight fast, provision of calories will reduce fat oxidation in both healthy subjects and weight-losing cancer patients (Selberg *et al*, 1990). Such an intervention would allow critical examination of the inter-relationships between circulating concentrations of leptin, interleukin-6 and fat oxidation during feeding in healthy subjects and weight-losing cancer patients.

## PATIENTS AND METHODS

Weight-losing cancer patients with an unequivocal diagnosis of pancreatic cancer were studied. No patient had clinical evidence of ascites, peripheral oedema, diabetes mellitus or malabsorption nor had undergone surgery, chemotherapy or radiotherapy in the preceding 4 weeks. No patient had clinical or radiological evidence of infection, were jaundiced or severely anaemic or were receiving steroids.

Healthy weight-stable subjects were studied as a control group.

### Study design

The study protocol was as previously described (Barber *et al*, 2000). Briefly, after an overnight fast, at 08:00 h baseline blood samples were collected for assessment of leptin, interleukin-6, insulin and cortisol. Following a rest period of at least 20 min baseline, oxygen consumption and carbon dioxide production were measured over a 20 min period using a ventilated hood indirect calorimeter (Deltatrac II, Datex, Helsinki, Finland). Resting energy expenditure was calculated as previously described (Barber *et al*, 2000). Healthy subjects and cancer patients received hourly meals (for 4 h) of a balanced whole protein liquid nutritional supplement (Fortisip, Nutricia, Zoetermeer, Holland), each meal providing one-twelfth of the estimated energy requirement (measured resting energy expenditure X 1.44). During the 4 h feeding period, the subject or patient continued to rest in the supine position and energy expenditure measurements were repeated every 40 min for 20 min and blood sampling was repeated every 30 min.

Urine was collected by healthy subjects and cancer patients for the 24 h period prior to attendance. The urine was collected over the 4 h study period to provide an estimate of urinary nitrogen excretion in the fed state.

The study was approved by the Research Ethics Committee of Edinburgh Royal Infirmary University NHS trust. All subjects were informed of the purpose and procedure of the study and gave written consent.

### Methods

Serum leptin was measured using a commercial kit (Human Leptin RIA kit; Linco Research Inc., St Charles, MO, USA). The limit of sensitivity was 0.5 *μ*g l^−1^, and the intra- and inter-assay coefficients of variation were 5.8 and 6.5%, respectively, over the sample concentration range.

Circulating concentrations of interleukin-6 were measured using a solid-phase sandwich enzyme-linked immunosorbent assay (ELISA, Diaclone Research, Bescanon, France). The lower level of detection was 2 *μ*g l^−1^ and the intra- and inter-assay variabilities were less than 4 and 6%, respectively, over the sample concentration range.

Circulating concentrations of insulin and cortisol were measured as previously described (Fearon *et al*, 1998).

Total body water was measured using a bioelectrical impedance system (Xitron 4000B; Xitron Technologies, San Diego, CA, USA). The error of the method is approximately 10% (Hannan *et al*, 1995). Body fat was calculated using the following formulae:

Fat-free mass (kg)=total body water/0.73

Body fat mass=weight−fat-free mass

Urinary nitrogen was estimated using a rapid combustion and thermal conductivity cell method (Leco FP-328, St Joseph, MI, USA).

Energy expenditure was measured by indirect calorimetry using a ventilated hood system (Deltatrac II, Datex, Helsinki, Finland). Coefficient of variation was less than 5%. Substrate utilisation was calculated from oxygen consumption and carbon dioxide production (provided by indirect calorimetry) and urinary N excretion in the fasting (baseline) and fed (after 200 min of the feeding protocol) states, as previously described (Barber *et al*, 2000).

### Statistics

Data are presented as the median and range. Where appropriate, serial data from time points during the feeding period were tested for statistical significance using the Friedman Rank test. Analysis between control and cancer groups was performed using the Mann–Whitney *U*-test. Statistical analysis was performed using SPSS software (SPSS Inc., Chicago, IL, USA).

## RESULTS

The characteristics of healthy subjects and patients are shown in Table 1

**Table 1:** Baseline characteristics of healthy subjects and weight losing cancer patients

	**Healthy subjects (*n*=6)**	**Cancer patients (*n*=7)**	
	**Median (range)**	**Median (range)**	***P*-value**
Sex (m/f)	3/3	3/4	1.000
Age (years)	54 (50–62)	59 (56–75)	0.018
BMI	26.3 (20.8–36.1)	20.4 (16.4–22.9)	0.010
Body fat mass (kg)	26.3 (16.8–40.3)	16.0 (12.6–20.1)	0.015
Weight loss (%)		15.9 (12.7–20.7)	
			
Insulin (mIU l^−1^)	6.2 (4.5–16.8)	4.2 (3.5–8.1)	0.063
Glucose (mmol l^−1^)	5.1 (4.5–5.6)	5.6 (4.4–6.1)	0.564
Cortisol (nmol l^−1^)	521 (382–626)	639 (437–677)	0.022
Leptin (*μ*g l^−1^)	12.9 (5.1–49.8)	2.1 (1.3–14.6)	0.032
Interleukin-6 (ng l^−1^)	<2 (<2–<2)	4.1 (<2–9.4)	0.024
			
Resting energy expenditure (kcal day^−1^)	1565 (1270–1940)	1425 (1035–1710)	0.199
Resting energy expenditure (kcal (kg body weight)^−1^ day^−1^)	20.2 (16.3–23.3)	24.3 (19.5–27.7)	0.046
Fat oxidation (mg min^−1^)	64.4 (39.4–83.0)	71.1 (20.4–109.3)	0.474
Fat oxidation (mg (kg body fat mass)^−1^ min^−1^)	2.44 (1.48–3.14)	4.73 (1.19–6.85)	0.032

. At baseline, compared with the healthy subjects, cancer patients had less weight and fat mass (*P*<0.05) and had lower circulating concentrations of leptin (*P*<0.05), but higher concentrations of cortisol and interleukin-6 (*P*<0.05). Resting energy expenditure and fat oxidation were greater in the cancer group (*P*<0.05).

Over the 4 h feeding period, insulin concentrations increased significantly both in the control (*P*=0.006) and cancer groups (*P*<0.001). In contrast, glucose concentrations remained stable in the control (*P*=0.318) and cancer groups (*P*=0.101). Cortisol concentrations fell significantly both in the control (*P*<0.001) and cancer groups (*P*<0.001). Leptin concentrations remained stable in the control (*P*=0.186) and cancer (*P*=0.062) groups. Similarly, in the cancer group with detectable interleukin-6 concentrations, they were not significantly altered (*P*=0.869) over the feeding period. Energy expenditure increased in the control (*P*=0.002) and cancer groups (*P*=0.003). In contrast, fat oxidation only fell in the cancer group (*P*=0.008).

The areas under the curve for the measured parameters are given in Table 2

**Table 2:** Feeding response of healthy subjects and weight-losing cancer patients

	**Healthy subjects (*n*=6)**	**Cancer patients (*n*=7)**	
	**Median (range)**	**Median (range)**	***P*-value**
*Area under the curve over the feeding period (200 min)*			
Insulin (IU l^−1^)	4.57 (2.82–9.05)	3.73 (2.72–7.26)	0.366
Glucose (mol l^−1^)	1.09 (0.94–1.20)	1.44 (1.15–1.76)	0.005
Cortisol (mmol l^−1^)	58.8 (52.8–81.3)	81.9 (65.3–128)	0.022
Leptin (mg l^−1^)	2.59 (0.94–9.97)	0.33 (0.22–2.69)	0.022
Interleukin-6 (ng l^−1^)	<2 (<2–<2)	753 (420–1554)	0.048

Energy expenditure (cal)	341.8 (285.8–435.3)	289.8 (212.4–355.0)	0.138
Energy expenditure (cal (kg body weight)^−1^)	4.61 (3.56–5.33)	5.14 (4.16–5.63)	0.101
Fat oxidation (g)	10.9 (8.1–12.5)	9.4 (2.4–18.1)	1.000
Fat oxidation (mg (kg body fat mass)^−1^)	380 (286–619)	678 (142–1036)	0.101

. Over the feeding period, the area under the curve for glucose, cortisol and interleukin-6 were greater in the cancer group compared with the control group (*P*<0.05), whereas leptin was significantly less for the cancer group (*P*<0.05). The areas under the curve for insulin, energy expenditure and fat oxidation were similar in both groups.

The molar ratio (molecular weights of leptin and interleukin-6 are 16 and 26 kDa, respectively) of the areas under the curve for leptin and interleukin-6 was approximately 1000-fold greater in the weight-losing cancer group (*P*<0.01).

## DISCUSSION

Few studies have examined the acute effect of feeding on circulating leptin concentrations in healthy subjects. Clapham *et al* (1997) reported no change in leptin concentrations in either adipose tissue or the plasma for 3 h following a mixed meal. Similarly, Dallongeville *et al* (1998) reported no change up until 6 h following a mixed meal. The results of the present study confirm these findings and would indicate that, in healthy subjects, circulating leptin concentrations are not altered acutely on feeding. To our knowledge, there have been no studies which have examined the acute effect of feeding in cancer patients.

In the present study, at baseline, weight-losing cancer patients had less fat, lower leptin concentrations and had increased concentrations of the catabolic mediators cortisol and interleukin-6. Despite a lower body weight and body fat mass, the cancer group had a higher resting energy expenditure and higher fat oxidation compared with the control group. Over the feeding period, the total amount of circulating leptin was less and the total amounts of glucose, cortisol and interleukin-6 were greater than that of the control group. Taken together, the results of the present small study would suggest that weight loss in pancreatic cancer patients is associated with an increase in catabolic mediators, insulin resistance and a reduction in leptin concentrations.

The clinical consequences of lower circulating concentrations of leptin and the greater concentrations of glucose, cortisol and interleukin-6 in cancer patients in the fed state are likely to be profound. These include continuing loss of lean and adipose tissue and the consequent decline in physical function (Fearon *et al*, 1999) and continuing impairment of the immune system and the consequent increase in infection (Fantuzzi and Faggioni, 2000).

Given their similar receptor activity, the reciprocal relationship between interleukin-6 and leptin is of interest. Indeed, between the control and cancer groups, there was a large increase in their molar ratio. Whether this is responsible for or secondary to the insulin resistance seen in the present study is not clear. However, an infusion of interleukin-6 has been shown to increase cortisol concentrations and fat oxidation in cancer patients (Stouthard *et al*, 1995). Furthermore, euglycaemic hyperinsulinaemic glucose-clamp studies have reported that insulin resistance increases with elevated circulating concentrations of interleukin-6 (Makino *et al*, 1998). These results would be consistent with observations that pancreatic tumour cell lines produce increased amounts of interleukin-6 (Wigmore *et al*, 2002).

If this were to be the case, then reduction of interleukin-6 concentrations would be a prerequisite for the reduction of insulin resistance and fat oxidation in weight-losing pancreatic cancer patients. Therefore, it is of interest that administration of fish oil has been shown to decrease interleukin-6 and cortisol concentrations and preserve fat tissue in these patients (Barber, 2001).

In summary, low circulating concentrations of leptin, increased fat oxidation and insulin resistance are associated with increased circulating concentrations of cortisol and interleukin-6 in weight-losing patients with pancreatic cancer.
